# The Impact of ERASMUS Exchanges on the Professional and Personal Development of Medical Students

**DOI:** 10.3390/ijerph182413312

**Published:** 2021-12-17

**Authors:** Paweł Żebryk, Piotr Przymuszała, Jan Krzysztof Nowak, Magdalena Cerbin-Koczorowska, Ryszard Marciniak, Helen Cameron

**Affiliations:** 1 Department of Medical Education, Poznan University of Medical Sciences, 60-806 Poznan, Poland; pzebryk@ump.edu.pl (P.Ż.); pprzymuszala@ump.edu.pl (P.P.); rmarcin@ump.edu.pl (R.M.); 2 Department of Paediatric Gastroenterology and Metabolic Diseases, Poznan University of Medical Sciences, 60-572 Poznan, Poland; jannowak@ump.edu.pl; 3 Aston Medical School, Aston University, Birmingham B4 7ET, UK; helen.cameron@aston.ac.uk

**Keywords:** ERASMUS program, student exchange program, medical students, professional development, medical curriculum, medical education

## Abstract

The ERASMUS program is one of the most popular student exchange projects, particularly among the students of Central and Eastern European countries. However, limited research is available with regard to its influence on the professional and personal development of its participants. The study aimed at investigating the experiences and impact of the ERASMUS program on different domains of the personal and professional life of medical students. A questionnaire containing closed and open-ended questions was distributed among 269 former participants of the ERASMUS program from the Poznan University of Medical Sciences to collect qualitative and quantitative data regarding the topic. The response rate was 41%. Mastering professional foreign language skills was the most frequently reported benefit of ERASMUS (94%), followed by a change of approach towards learning by exposure to innovative teaching techniques, character, professionalism and cultural competency development, impact on the migration decisions of the students, as well as the opportunity to compare healthcare and educational systems across countries. Additionally, 57% of respondents stated that ERASMUS impacted their career plans, and few indicated that it had affected their specialty choice. Approximately 28% of respondents have worked abroad in healthcare or research since graduating. Participation in the ERASMUS program proved to be a unique opportunity for professional and personal development.

## 1. Introduction

The ERASMUS (European Community Action Scheme for the Mobility of University Students) program was founded in 1987 by the European Commission, and its purpose was to enable cooperation and unification of higher education across European countries (which comprise European Union (EU) members, countries applying for EU membership, as well as European Economic Area members). The goals of the program are achieved mainly by means of enabling and co-financing the international mobility of students and academic workers between European countries [1].

The Bologna Process was launched in 1999 to ensure comparability in the standards and quality of higher education qualifications, as well as to promote mobility and cooperation of the staff and students at a European level. With the introduction of the European Credit Transfer and Accumulation System (ECTS), student exchange was facilitated due to the mutual recognition of academic achievements across different European higher education schools [1].

The idea of the ERASMUS program is that a student of one European academic institution spends part of their study in the institution of a foreign European country for a period ranging between three months and one academic year. The student joins other students in the host institution in their courses, and is subjected to the host’s standard requirements for each of the courses followed. The exchange is made possible by bilateral agreements between the host and home institutions, part of which is that schools will mutually recognize all training and assessment the exchange student undertakes. In some host institutions, the exchange students join native students and learn in their mother tongue, whereas in others (particularly where the mother tongue is not commonly spoken worldwide), students either learn in divisions that teach medicine in English or are assigned a student interpreter/guide. Furthermore, in order to make ERASMUS exchanges possible for all countries despite economic discrepancies, the exchange students receive a monthly subsidy which helps to compensate for the differences in the costs of living between the host and home countries [2].

Benefits of the ERASMUS and other student exchange programs are widely recognized by their participants and include personal and professional development, experience of other cultures and a change of surroundings, language skills improvement, traveling, as well as a boost in self-confidence [3,4,5,6]. Medical students additionally value them for the opportunity to practice their interpersonal skills with patients from different cultural backgrounds, improve their clinical skills, to be exposed to the local health problems which are rarely observed in their homelands, and compare different medical education and healthcare solutions with their local systems [7,8]. The possibility of spending part of their studies in another country seems to be particularly appealing to citizens of the Central and Eastern European countries, and Polish participation is noted for being particularly dynamic [9].

However, despite the significance of the program and the substantial interest of students and authorities, detailed research on the impact of ERASMUS on medical students’ professional and personal development remains limited. Stakeholders, such as the potential ERASMUS medical students, the European Commission, and deans of the medical faculties are keen to establish whether the significant economic and organizational effort required in arranging ERASMUS exchanges yields worthwhile educational and cultural profit. Consequently, the aim of this study was to explore the experiences of Polish medical students participating in the ERASMUS program in detail. The focus of the study comprised several domains of personal and professional development, including education, language skills, career, migration and learning about healthcare delivery systems.

## 2. Materials and Methods

The study was based on the mixed method which adopted a general inductive approach to data analysis to provide greater insight into the issues through both quantitative and qualitative data [10,11].

Ethical approval was obtained from the Bioethics Commission at the Poznan University of Medical Sciences (Resolution No. 528/12), since the potential participants of the study were either former or current students at this university.

Following a literature review, an Internet-based questionnaire with closed and open-ended questions was designed in English using the brainstorming technique, and its final version was translated by the authors into Polish. Three independent Polish native speakers with excellent command of English checked the integrity of the translation and trialed the questionnaire. The questionnaire was constructed with several domains in mind: career, migration, language, cultural, social, educational, and healthcare in order to grasp the depth and exchange transformative potential of ERASMUS experience. The questionnaire comprised 35 questions, including 24 closed and 11 open-ended ones. In the rating questions, a 5-point Likert scale was used. The detailed outline of questions, as well as the number of participants responding to each question is presented in Appendix A Table A1.

A list of email addresses of all medical students of the Poznan University of Medical Sciences (PUMS) who participated in the ERASMUS program was provided by PUMS. An email invitation to complete the anonymous questionnaire was sent out to the former ERASMUS participants using Surveymonkey.com (Palo Alto, CA, USA) with two reminders. All emails included an option to opt-out of any further messages and emphasized the anonymous nature of the study. The questionnaire additionally provided an explanation of the project, including the assertion regarding its anonymity, confidentiality, and the ability to withdraw before submitting it. The authors used implied consent to the research.

The questionnaire was circulated among the target population of 269 former and current medical students in May and June 2012. All the respondents undertook a period of study (between three months and one year) at a foreign institution within the ERASMUS program between the years 2000 and 2012.

Quantitative data were analyzed with the STATISTICA 13 statistical software package (StatSoft Inc., Tulsa, OK, USA) using descriptive statistics, Wilcoxon signed-rank test, Spearman correlation and χ^2^. Likert scale intervals were designed to be equal for all the questions where it was employed. The qualitative content was subject to a thematic analysis with the aid of N-vivo 9 software 2010 (QSR International Pty Ltd., Doncaster, Australia). In the process of rigorous and analytic reading, open codes emerged and were further classified into axial codes. Coding was performed by P.Ż. and was independently checked by H.C. and J.K.N. to ensure validity, as well as to reduce bias.

## 3. Results

### 3.1. Study Population

The response rate of the questionnaire was 41% (109 out of 269). A variety of participant characteristics was observed regarding their age (range 22–36), gender (25% male, 75% female), and current professional role (including doctors working in various healthcare settings, members of the academic staff, PhD and medical students).

**Figure 1:**
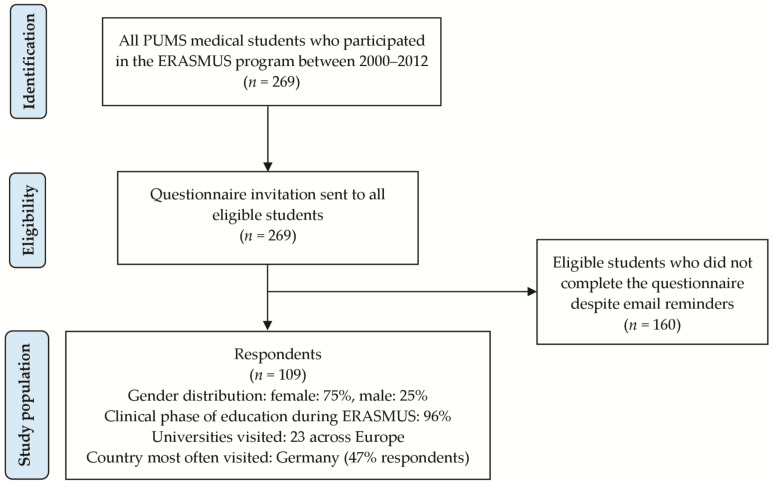
Flowchart detailing the enrolment process and the study population characteristics.

The period during which participants undertook the ERASMUS program lasted from 2000 to 2012, with increasing number of respondents towards more recent years of participation. Almost all respondents participated in ERASMUS during the clinical stage of education, and they visited one of 23 universities across Europe, with Germany as an unequivocal leader (47%), followed by France (9%), Spain (7%), the Netherlands (7%), Portugal (6%), Sweden (6%), Finland (6%), Hungary (5%), Turkey (3%), Italy (3%), Denmark (1%), and the Czech Republic (1%). The enrolment process was depicted in a flowchart (Figure 1).

### 3.2. Motivation

The participants were asked to select all the reasons for going to ERASMUS from a predefined list. The most common reasons included using the existing opportunity, a chance to improve language skills, experiencing a different culture, and exploring a different educational system. The detailed characteristics of the collected responses was presented in Table 1.

### 3.3. Language Experience and Impact

An improvement in the professional foreign language skills was the most frequently reported benefit of the participation in the ERASMUS program, indicated by 94% of participants. Specifically, 77% of the participants considered these skills improved significantly, 17% stated they improved a little, with only 6% observing no change (χ^2^ *p* < 0.001).

**Figure 2:**
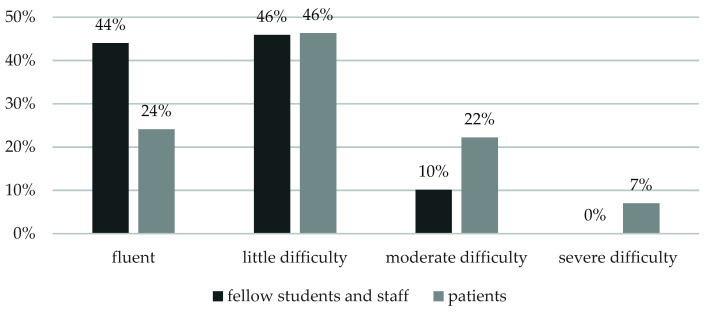
The participants’ declared competence in communicating with patients vs. fellow students and the staff in the host institution.

Additionally, 87% of the respondents indicated language as an important factor in choosing the ERASMUS destination, and 90% of them preferred to improve an already known language, with only 10% of the participants deciding to learn a new one. Although most students confirmed communicating with fellow students/academic staff and patients using the host country language (71% and 73%, respectively), communication with patients turned out to generate more difficulties (Figure 2).

In two reported cases, the academic staff used their native language despite the fact their students did not understand it:

*“Doctors often did not feel like speaking English, they spoke Dutch knowing that I could not understand it at all. It was also mandatory for me to participate in the meetings held in Dutch, which I could not understand. The classes lasted a very long time and often were not very educational. I had to stand in the operating room and not interrupt anybody. (...)”* (P003)

The practice of excluding the exchange students through language was reported as an important issue by some respondents:

*“During my neurology classes […] the tutors generally did not care that they have ERASMUS students in the group and did not even bother to ask whether something needs to be repeated or translated into English (certainly a negative experience).”* (P023)

### 3.4. Learning Experience and Impact

Participation in the ERASMUS program also allowed the students to compare the education systems in Poland and in the host country. The thematic analysis of their insights on these differences was presented in Table 2.

Furthermore, 90% (71/79) of the respondents indicated that they would like to see changes introduced to the Polish medical education system, although 4% (3/79) claimed it was irrespective of their ERASMUS experience. On the other hand, 10% (8/79) said they would not like the Polish curriculum to change. A number of ideas to be included in the Polish medical education system were suggested by the respondents, such as allowing and expecting Polish students to be more active in the learning process, providing them with the responsibility for patient care in the healthcare team, and focusing their learning on attaining higher levels in Bloom’s taxonomy. Moreover, students believed that their teachers should demonstrate more respect towards them, have time off duty while teaching, and be recognized for the quality of teaching. In addition, horizontal planning between courses should also be introduced to eliminate the existing knowledge redundancy. The abovementioned points, raised by several students, were clearly articulated by one of them in the following manner:

*“We need:*

- *To enable (incorporate) students to patient care*
- *to clearly define expectations for students in the wards*
- *to introduce student night duties (sign-up lists, one student per duty, full night shifts)*
- *case-based teaching*
- *more freedom to choose classes (it does not make sense to force students to attend bad lectures. They will attend good ones without obligation if it corresponds to the exams or due to the pressure of the social environment in the culture of responsibility)*
- *long-term strategy for improving the culture of professionalism and unity of the profession (i.e., by shared meals) greater emphasis on practical training, i.e., its intellectual, interpersonal as well as manual components*
- *a change in the assessment of knowledge from the MCQ tests to problem-solving and consequently reorientation of efforts by students*
- *elimination of requirements for detailed facts, but strict enforcement of basic skills and knowledge*
- *creating an environment in which physicians will have more time for themselves and for students–i.e., working one full shift time should be enough to live well, without having to work second and third jobs*
- *conduct a broad assessment of the quality of teaching and learning and drawing the consequences upon its results.”* (P108).

In terms of the post-graduate education, the participants emphasized that an open-access and flexible specialty training system should be established.

*“Possibility to choose any specialization regardless of the LEP [state exam] grade, better earnings during specialization. Possibility to change specialization. (…)”* (P061)

Moreover, 62% of the respondents (46/74) reported that the ERASMUS experience had changed their approach to learning. Among the most notable changes, the students indicated, e.g., focusing on the clinical, practical, and important, practice of evidence-based medicine, as well as learning to understand instead of crude memorizing:

*“I definitely changed my approach to learning. This exchange opened my eyes. My studying has become more focused, effective, less rote learning, more understanding, and correlation. Paying attention to the really important stuff, not some test nuances.”* (P059)

*“Yes. I learned how to ‘learn’. Instead of memorizing drug doses by heart, I am now trying to remember where they are used. Learning doses will finally occur naturally. The same goes for the symptoms of diseases, diagnostic approach, and differentiation.”* (P080)

In general, it seems that because of the ERASMUS experience, the approach to learning has matured in a number of students. The thematic analysis of content revealed various ways in which the change might have occurred (Table 3).

**Figure 3:**
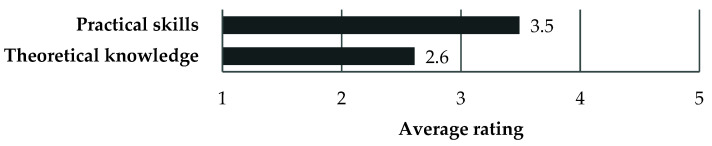
The respondents’ average assessment of the competencies of the students from the host institutions compared with the Polish counterparts.

Interestingly, the participants (*n* = 102) assessed the practical skills of students from the host institution higher (3.5 ± 1.1 (median = 3)) on a 1–5 Likert scale, whereas the theoretical knowledge was assessed lower (2.6 ± 0.8 (median = 3)) in comparison with the competencies of Polish students. The difference in comparison between practical skills and theoretical knowledge was statistically significant (*p* < 0.001) (Figure 3).

Some participants also reported developing self-directed learning skills:

*“Studying has become a little more regular, a little more focused on practice. I paid a little less attention to the Polish school demands if I reckoned they were not right (...).”* (P108)

*“I learned an education system in which it is up to you what you get out of it and not that you are doing something because you are constantly controlled and tested.”* (P057)

However, some respondents stated that they could not sustainably change their learning style following ERASMUS due to the requirements and the predominant learning culture of the home institution.

*“[changing approach to learning] was difficult, because of the requirements of the home institution are different (emphasis on theory) (…).”* (P037)

### 3.5. Healthcare and Clinical Practice Experience and Impact

During the ERASMUS program, students could also compare the healthcare systems between the visited country and Poland. Among 65 students who elaborated on the topic in response to an open question, only 10 stated there were no significant differences. The rest reported differences which pertain to a different organization, culture, and financing of healthcare. The thematic analysis of their responses is presented in Table 4.

A nearly equal number of participants stated their approach to clinical practice changed (*n* = 32), or did not change (*n* = 29) following the participation in the ERASMUS program. The respondents who observed a change, most frequently indicated it in the area of soft skills, such as doctor-patient communication. Several study participants emphasized the observed professionalism in the approach to patients as one of the most influential factors modifying their future medical practice:

*“Yes, especially the approach to patients [has changed]. I was taught to respect the patient very, very much, always introduce myself and address them with impeccable manner, regardless of the situation.”* (P063)

*“During my clinical courses in the hospital, sometimes patients were homeless. Watching the doctors who treated them with great respect equal to other patients, I have definitely learned that every patient is equal, and everyone must be helped as best as we can regardless of the social or material status.”* (P029)

Most participants (72%) admitted they recognized the need for a change in the Polish healthcare, another 10% also agreed, although their opinion was not shaped by ERASMUS, and only 6% saw no need for a change. A small group of respondents expressed their doubts with regard to the reality of bringing about a change or questioned its affordability bearing in mind Polish gross domestic product (GDP) per capita. Yet another participant feared that politicians who control the healthcare system would change the system towards new restrictions and extensive control. In addition to the implementation of the healthcare solutions listed in Table 4, the students suggested other changes which could be introduced in Poland, including flattening the medical hierarchy, shifting the burden of paperwork from doctors to medical secretaries, reimbursement of costs of continuing education for doctors, mandatory courses on doctor-patient communication, and a module specialty training leading to faster professional independence.

### 3.6. Career and Professional Migration Experience and Impact

The proportion of respondents who acknowledged that the ERASMUS exchange influenced their career plans was 57%. Approximately 28% of the study participants reported working abroad in healthcare or research after the ERASMUS program. The average ratings (1—less, 5—more) of the impact of ERASMUS on respondents’ plans to look for a permanent job abroad and undertake post-graduate studying and training abroad were 3.8 ± 1.2 (median = 4) and 4.0 ± 1.1 (median = 4), respectively, and they were highly correlated (Spearman’s R = 0.81, *p* < 0.001).

In the course of the application for the program, more respondents perceived ERASMUS as a chance to improve their prospects of getting a job abroad (44%) instead of Poland (17%).

A relatively small number of participants listed additional research opportunities during ERASMUS (7%). Nevertheless, those who did, appreciated the opportunity of pursuing professional doctorates in the course of undergraduate studies.

### 3.7. Personal and Social Experience and Impact

Apart from the purely educational and professional gains, the respondents frequently reported various personal and social benefits from ERASMUS:

*“I think that the most important benefits of participation in such a program are absolutely immeasurable and impossible to describe in any survey because they are mainly related to the evolution of consciousness, character, and horizons. I believe every student should have the opportunity to participate in this or any other form of exchange.”* (P034)

**Figure 4:**
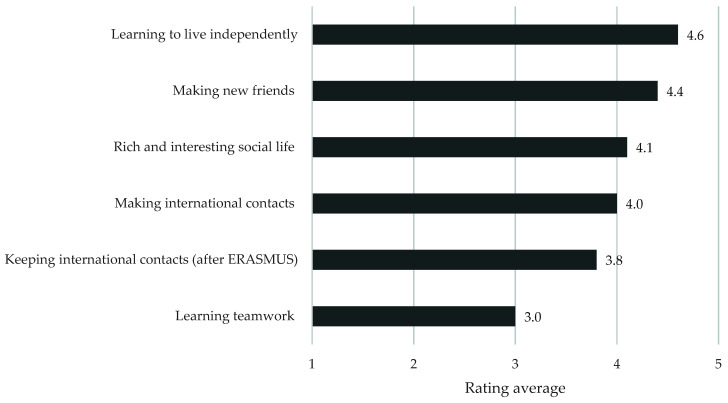
The respondents’ evaluation of importance of personal and social benefits gained from the ERASMUS program (Likert 1–5). SD was in the 0.7–1.2 range.

The ERASMUS participants evaluated learning independence, making friends, and international contacts highly, as presented in Figure 4.

### 3.8. ERASMUS as a Catalyst of Change?

In response to the question whether they had tried to introduce changes in the Polish education or healthcare system because of the ERASMUS program, 104 respondents answered this question (“yes” *n* = 23, “no” *n* = 81); and 5 omitted it. Of the 23 participants who admitted trying to implement changes to the Polish educational or healthcare systems, 19 elaborated on the outcomes of their efforts. The analysis of the responses (Table 5) revealed limited or no effect of the former ERASMUS exchange students trying to implement changes in their professional circles, at times faced with a negative response or plain indifference. This, in turn, led to frustration, and some participants admitted ceasing to convince others to change.

In order to assure the relevance of answers to the open questions, the study method assumed that all the unique information collected was relevant for the characterization of the investigated phenomena. Therefore, the answers to the open-ended questions were coded, as described in the methods section. However, it should be noted that the relevance of results of their analysis might not be equal for each question. In fact, it depended on the number of the received responses, the quality of descriptions and appearance of a persistent pattern across responses, as well as on the concordance with the closed questions results. Nevertheless, with most questions a clear pattern was visible even with a smaller number of responses, and this was consistent with the findings from the closed questions.

## 4. Discussion

Despite the almost 35-year history of the ERASMUS program, the research with regard to its impact on the professional and personal development of medical students is scarce. Most of the studies published to date which concerned the medical student exchange [7,8,12,13] have addressed the short-term experiences abroad in the so-called international health electives (IHE), where students from developed countries visit developing countries for a period from two weeks up to three months [12]. Jeffrey et al. [8], in their systematic literature review concerning the medical student short-term IHE, found that they offer substantial opportunities to learn new, or to improve the existing diagnostic and clinical reasoning skills, particularly in history taking and physical examination in a setting with limited access to high-tech hospital equipment.

In contrast, this study aimed to explore the range of experiences of medical students participating in the ERASMUS program, as well as how the exchange affected them professionally and personally. The study was extensive, using a questionnaire designed to capture both quantitative and qualitative data. Considering the time-lag since the participants had undertaken their exchanges, the response rate of 41% was surprisingly high, and even more satisfying bearing in mind the fact that the detailed and comprehensive answers had been provided for the open-ended questions.

In terms of gender distribution of the respondent population, as shown by Böttcher et al. [14], female students seem to be generally over-represented in the ERASMUS program. Moreover, according to the Polish General Medical Council (GMC), men comprise 37% and women 63% of doctors under the age of 35 [15]. Although gender distribution of young doctors partially explains the female predominance in the study population, it is difficult to account for its scale due to insufficient data.

The distribution of countries visited by the participants seems to be mainly a derivative of the bilateral agreements with partner universities. For instance, the policy at German universities is very open for incoming students, and a number of scholarships are available. A study conducted by Kumwenda et al. [16] demonstrated that student destination choices might be shaped by the expected costs and available scholarships. Their choices may also be affected by tourist attractiveness, geographical proximity, and popularity of the language spoken in their destination country [17]. Germany was also the main destination of Polish students in the study by Bryla [9] with the result of 11.0%, followed by Spain (7.6%) and France (7.3%), and the fact that the top three countries were comparable in both studies seems to justify the aforementioned assumptions.

The role of money was not the focus of this research; nevertheless, it is potentially significant as indicated in the previous studies [18]. A subsidy for ERASMUS does not cover all the costs of living abroad. Hence, a plausible explanation for the lack of students raising this as the serious problem is that those who went had enough resources, whereas those who did not have the funds simply did not even contemplate the exchange. In 2004/2005, among the ERASMUS participants from Poland, 48% came from families of high or very high income [19]. Furthermore, over half of the program participants knew some students who were deterred from the program due to financial reasons [19]. In a study conducted by Goodman et al. [20], funding was also recognized as a barrier to studying abroad. However, students’ knowledge and awareness of the existing funding seem to be limited, although they might constitute important factors in their interest and decisions regarding the participation in the exchange programs [18]. Thus, university authorities could explore this issue further in order to promote equity and broader access to this opportunity.

Among the benefits of the participation in the ERASMUS program, language, personal and social benefits were, in general, assessed higher in terms of importance than educational benefits, yet all were rated above average. This is consistent with the available literature, as students’ motives for participating in exchange programs seem to be more personally-oriented than strictly professional [17]. Additionally, Teichler et al. listed relaxation and vacation as an inspiration for two-thirds of ERASMUS students, and similar results recurred in several other studies. However, this does not necessarily contradict the objectives of the ERASMUS program [3,4,5]. In fact, after exposition to other cultures and the possibility to reduce stress, students may be more focused on professional and educational development upon their return home, thus, positively affecting them in the long run [17,21].

Furthermore, language also constituted a barrier for some participants, particularly those unfamiliar with the mother tongue of the visited country. For instance, some students were required to participate in events held in a language which was completely unknown to them, even though the organizers must have been aware of that. Hence, this seems to be a vital issue to be addressed by the host universities, since such situations should not occur. Interestingly, a similar observation was made by a group of midwifery students from the United Kingdom who visited Malta and found themselves in situations when the staff were speaking Maltese in their presence, initially making them feel excluded [22]. Another observation was a difference in the perceived difficulty of communicating with patients compared with the staff and fellow students. Presumably, the communication with the highly educated university staff or students was easier than with the patients, as the latter presumably had different educational backgrounds, may have used dialects of their mother tongue, and may not have spoken English fluently. Our results seem to be mirrored by Keogh et al. [6]. In their study, communicating with patients due to the language barrier also constituted an obstacle, although fewer difficulties were reported in communicating well with the staff. A literature review conducted by Brown et al. [18] also emphasizes the importance of language skills as a factor discouraging students from studying abroad and demonstrates the dependence of the student destination choice on the language spoken in the visited country.

The differences in medical education between Poland and ERASMUS host countries were abundant. The most notable discrepancies were found in terms of the culture of teaching and learning, as well as in the approaches to the curriculum. The participants also positively evaluated the fact that students in the host institutions adopt more responsibility both for their learning, as well as within the healthcare team. In contrast, in Poland, teachers still take a more significant role in managing the learning process. They require knowledge from students, and the aforementioned pressure and controlled motivation is hardly a good foundation for life-long learning. On the other hand, even though the requirements were much more liberal in the host institutions, students were aware of the reasons as to why they had to learn, and were autonomously motivated according to the self-determination theory (SDT) [23]. Similarly, in the healthcare setting, students in the host institutions were frequently responsible for patient care and constituted important assets in the hospitals. Conversely, in Poland, classes often resemble “guided tours”, according to one respondent, with clinicians, but devoid of any real responsibility and engagement. Therefore, it would seem that teaching in the visited institutions frequently fostered self-determination. In most institutions, the participants reported the emphasis on the clinical, practical, and important, and this slogan could serve as a general principle of the way curricula were organized. Students were not required to know numerous theoretical details and irrelevant information, but instead were meticulously taught practical and absolutely core material. This contrasts with the respondents’ experiences in Poland; in fact, the emphasis is just the opposite. Individual learning with one doctor was frequently stated as a fundamental method of organizing clinical courses. In Poland, the traditional groups of six students per doctor are one of the biggest challenges of the medical education, since real work-based learning is impossible with so many students. This, in turn, renders it difficult to provide students with autonomy for their learning, or a sense of responsibility and importance in the clinical team, and hence they do not develop self-determination [24].

Moreover, it was interesting to observe the differences in the evaluation of practical skills and theoretical knowledge between students from the host institution and the Polish medical students. This generally confirms the findings according to which the educational systems of the host universities usually emphasized clinical training over extensive theoretical knowledge. The vast majority of the participants wished to implement changes to the Polish education system in order to adapt to the modern teaching methods. However, large-scale reforms in institutions, such as universities characterized by the structural and behavioral inertia, are challenging and time-consuming [25,26], as well as might require a generational change [26]. Therefore, it was not surprising that few respondents in our study attempted to introduce institutional changes. However, a change at a more personal level was reported by 2/3 of the respondents who stated that the ERASMUS experience had changed their approach to learning. As pointed as by some participants, they could not alter their learning style due to the home institution’s requirements and its predominant learning culture. This seems to be an interesting area for further research.

In addition, the study participants observed numerous differences in healthcare systems between Poland and the visited country, which allowed them to compare both systems and identify the potential areas for improvement in the organization of the Polish healthcare. Experience of ERASMUS among nursing students was documented in the qualitative study of seven former German-Finnish exchange students by Keogh et al. [6]. The authors also found that ERASMUS allowed students to experience a different healthcare sector, thus enabling a critical look at the home system, changing the approach to one’s own nursing practice and encouraging students to acquire new clinical competencies stemming from good teacher supervision.

Approximately 28% of the study participants reported working abroad in healthcare or research, compared with 7% of the total number of doctors who obtained a certificate of qualification to practice medicine in the EU from the Polish GMC. This measure is widely used in Poland as a rough estimate of the number of doctors employed abroad, due to the lack of more precise statistical data [27]. Thus, it would be tempting to argue that there is a causal connection between the ERASMUS experience and the willingness of students to migrate. However, a more thorough exploration of this issue would be necessary. Kolanowska demonstrated that more than 90% of Polish participants of the ERASMUS program consider it as a helpful factor in their future professional life [28], and its importance in that field was also evaluated in a study by Bracht et al. (the VALERA project) [29]. They investigated the professional value of ERASMUS by surveying former ERASMUS students (more than 4500) from various disciplines, as well as teachers, university leaders, and employers. One of the remarkable findings was a substantially higher self-reported professional value of ERASMUS for the former students and teachers from Central and Eastern Europe compared to their Western European peers. Furthermore, former ERASMUS students were more likely to be employed in a different country than the one they graduated from at any time after graduation (18–20% vs. 3% average among Europeans with similar qualifications). They tended to spend less time searching for a job and to find one more quickly than the non-mobile students. Unfortunately, even though the cited study was based on a large population of students in different disciplines, it was quantitative and rather generic; hence, it did not investigate the experiences of the medical students in detail.

We also acknowledge the limitations of this study. It was a single-center study which perceived the ERASMUS from the perspective of Polish medical students and involved only the outgoing students. Additionally, there may have been an element of selection bias to the study, i.e., those who responded to the questionnaire invitation may have had strong feelings about ERASMUS. Since the study participants were from one center in Poland, it does not address whether students from other European countries, both Central and Western European, or the incoming students in Poland, share similar experiences concerning the ERASMUS program. Future research should also attempt to address more recent experiences of ERASMUS participants from 2012 to 2021.

It should be considered that the qualitative aspect of the study may have led to some selection bias, and that not all relevant phenomena were revealed and investigated. Representativeness of the responses may be limited (despite sampling from a quite homogenous group), but they are investigated to conceptually characterize some mechanisms with sound, logical potential of generalization. Therefore, the quantitative side is complemented by the qualitative questions. The percentage of answers to the open-ended questions may be considered relatively high, based on the authors experience with conducting surveys in this community over the past decade. Moreover, in many areas we have seen a concordance in what was revealed by the qualitative and quantitative elements of the survey.

## 5. Conclusions

Stefan Wolff coined the term “the ERASMUS generation” with regard to the vast group of the former exchange students who are multilingual, multicultural, work across the European continent, and claim European identity [30]. This study demonstrates that ERASMUS provides Polish medical students with significant opportunities for further professional and personal development, particularly through language skills, the development of student-centered approaches to learning, and a shift in clinical practice attitudes from paternalistic to patient-centered. The abovementioned factors have created a considerable educational profit from participating in the ERASMUS program. Additionally, the participation in the ERASMUS program also allowed the respondents to learn the differences between medical education systems in Poland and host countries, especially the culture of teaching and learning and the approaches to the curriculum. Similarly, it also constituted an opportunity to observe differences and compare healthcare systems, including the organization, culture, and financing of healthcare. The ERASMUS experience impacted career plans of 57% of respondents, and 28% of them reported working abroad in healthcare or in research afterwards. The conducted study suggests that the benefits of ERASMUS far outweigh the few reported negative experiences related to cultural intolerance or insensitivity of some teachers and students, yet clearly there is still more to be done with regard to encouraging equality, diversity, and inclusion.

## Figures and Tables

**Table 1:** The reasons the participants selected for taking part in the ERASMUS exchange.

Category	Reason	% (*n* = 109)
Language	to improve language skills	85
Educational	to explore a different educational system	69
	to improve the quality of my education	61
	to improve my prospects of further study abroad	35
	to undertake the specific courses offered	22
	for research opportunities	7
	to improve my prospects of further study in Poland	7
Personal	to use the existing opportunity	88
	out of curiosity	55
	to improve my prospects of getting a job abroad	44
	for a change of routine	40
	to improve my prospects of getting a job in Poland	17
	for personal reasons	15
Social	experience a different culture	72
	to lead a more interesting social life	41

**Table 2:** Respondents’ observations upon major differences in the educational systems in the host country as compared with Poland.

Axial Code	Theme	Exemplary Quote
Professionalism	Doctors’ and students’ professional conduct towards patients	*“Doctors’ and medical students’ approach to patients was very professional and showed impeccable manners.”* (P036)
Culture of teaching and learning	Student-teacher partnership	*“Students are partners for lecturers, not ‘pupils’.”* (P084)
	Students have responsibility	*“In the University I was on ERASMUS much attention is paid to students’ self-study”* (P005) *“Students in the clinical part of the training have much more responsibilities than the Polish intern”* (P046)
Approaches to the curriculum	Emphasis on the clinical, practical and important	*“(…) German students, even though they study less extensively, have much better knowledge of the essentials. They learn practical and important things.”* (P005) *“All students can draw blood, put in intravenous lines, change dressings, which is a rare thing among students in Poland”* (P076)
	Individual teaching	*“Students usually follow the doctor one by one and take over some of the doctors’ duties, therefore, helping them.”* (P005)
	Flexibility	*“Less rigorous attendance check. Student takes responsibility for their education and should be conscious of its purpose and importance”* (P094)
	Case-based learning	*“More emphasis on practice: seminars in the form of Case-based Learning”* (P023)
	Practice of evidence-based medicine	*“Preparing to work in the principles of evidence-based medicine: searching articles on certain clinical topics and discussing them in a presentation with grading the level of evidence”* (P009)
	Assessment reflecting the practical and important knowledge and skills	*“Exams rely upon solving clinical cases, which, in my opinion, better prepares for future practice.”* (P041)
Modern facilities	E-learning platform	*“[university name] has an e-learning platform. Departments make educational and exam-relevant content available online (...) For example, Department of Orthopaedics and Neurology posted educational videos from physical examination, X-rays, MRI, CT interpretations, etc.”* (P029)
	Good medical textbooks specifically designed for medical students	*“Students have good textbooks–something like our state-exam preparatory textbooks, which explain plain and short the most important material”* (P040)
Internship	The final year of medical school similar to the internship in Poland	*“Final 6th year of studies similar to Polish internship”* (P069)

**Table 3:** Themes emerging from the content analysis of the respondents’ description of the effect the ERASMUS exchange exerted on their approach to learning.

Axial Code	Themes	Exemplary Quote
Learning style	Emphasis on the clinical, practical, and important	*“Certainly, I understood that you need to learn important things that one will remember in the future and not everything a medical textbook for a given specialty contains.”* (P057)
	Learning focused on attaining higher levels in Bloom’s taxonomy	*“I try not to focus on the details. I learn so that I can put the acquired knowledge into practice.”* (P076)
	Less stressful attitude to learning and assessment in Poland	*“Yes, the grades are not as important to me as they were before the exchange.”* (P050)
	Practice of evidence-based medicine	*“Yes. I started to learn more algorithms and based on current reports (articles) than textbooks.”* (P063)
	Learning from foreign textbooks	*“Yes, I focused on the essentials and started learning from German and English textbooks.”* (P052)
	Self-directed learning	*“I learned distance to exams. I began to learn more for myself and pay more attention to long term retention of what I learned.”* (P069)
	Emphasis on professionalism	*“It was difficult because the requirements of the home institution are different (emphasis on theory), but [the ERASMUS] definitely changed my approach to the patient, nowadays I pay more attention to it.”* (P037)
	Developing research interest	*“The Erasmus program allowed me to further develop and work abroad. It is very easy to start a doctoral thesis, both research and retrospective.”* (P002)
Personal	Balancing studying with spare time activities	*“Studying for four years in Poznan, I sacrificed most of my time studying. (…) Most people have no interests/hobbies outside of medicine. Their full attention and time are focused on exams. Here [in the host institution], it looks completely different. (…) Many people have their passions, which they constantly develop. Certainly, after the ERASMUS, I will try to change the way of learning. I would try to develop my interests, better organize my time off and balance it with studying, just as I managed to do here.”* (P029)
	Broadening horizons	*“Maybe not towards learning, but I expanded my worldview more and changed my approach to the patient”* (P018)
Unchanged approach	Change impeded by the Polish medical education system and its predominant culture	*“Of course, I regret that after returning to Poland I will have to study again as befits a Polish medical student and I will certainly have less time for non-scientific activities”* (P079)
	Unchanged: always strived to learn the best one can	*“Rather not, I try to learn the best I can.”* (P044)

**Table 4:** Participants’ observations regarding the major differences between the healthcare system in the host country and in Poland.

Axial Code	Theme	Exemplary Quote
Modern healthcare	Patient-centered and friendly healthcare	*“Yes, more attention is paid to the doctor-patient relationship.”* (P037)
	Hospitals well equipped and comfortable	*“Hospitals are better equipped, more diagnostic tests are available without waiting in the queue”* (P053)
	GPs have a central role in the system	*“A GP has more power, and greater skills. In Poland, a GP’s work often comes down to the extension of chronic prescriptions and referrals to specialists.”* (P037)
	Well-developed outpatient care	*“A more extensive outpatient clinic system. A system focused on the effectiveness of its activities, less willing to admit patients to the ward. A very fashionable system of one-day admissions, performing minor procedures in outpatient conditions, e.g., tonsillectomy.”* (P080)
Professionalism and culture	Doctor-patient partnership	*“(...) In Germany, I noticed a much smaller distance between a doctor and a patient-they are on almost equal terms. In Poland we’ve seen many doctors who behave as if they were “gods”. On the other hand, I also noticed much more mutual respect. Perhaps due to the fact that we do not respect patients they do not respect us?”* (P072)
Training	Junior doctors have independence and access to good clinical training	*“Junior doctors even in large clinical hospitals are from the onset of their career allowed to do many medical procedures or operate and have large independence in their work. In Poland, a junior doctor frequently does not have such independence.”* (P059)
Finance and insurance	Significantly more financial resources in the system	*“The French healthcare system, being financed much better than the Polish one, offers patients much more high-quality services. Modern drugs, diagnostic procedures, relatively short waiting times-are common.”* (P108)
	Coexistence of state or private insurance	*“In Germany, in addition to the state insurance, there is a widely available private insurance system. Almost every hospital has dedicated parts of wards for private patients. Queues are much shorter there or even none. (…) In Poland, on the other hand, everything for everyone”* (P072)
	The liability of health insurance clearly defined	*“In Poland, it is not defined what is covered by the national insurance. Refusals of financing certain treatments by hospitals are always met with astonishment [among patients]. In Germany, the financing is different. There are well-defined limits of coverage (liability), and if someone has more expectations, they can always change their insurance. (…) The rules are clearly defined.”* (P018)
	Rational allocation of resources	*“Reasonable taking care of finances: if in a given case two therapy options can be used with similar effectiveness-the cheaper one is chosen-thanks to this when the patient really requires expensive treatment-there is no waiting with its implementation to check if the “standard therapy” will not work. (…)”* (P034)
Information flow	Comprehensive electronic patient health records	*“From the first day of class, I admired the complete integrated system of patient data. All procedures, diagnostic tests, consultations, hospital stays, outpatient visits are stored in one computer system, accessible to all doctors after logging in.”* (P005)
	Thorough patient information on the disease, therapy, and plans	*“I like the detailed patient information on the procedures they are subjected to. Each patient receives a well-prepared printed detailed description of the procedure and possible side effects. This facilitates cooperation with the patient.”* (P005)
	Consultations with the doctors the patient attended before	*“In addition, in hospitals, doctors frequently consult on the phone with a doctor under whose care the patient was before. This avoids double testing and accelerates the acquisition of information about the patient.”* (P005)
Management	Better organized and managed healthcare	*“Doctors work in better conditions, they have more time to deal with patients, salaries are higher and therefore they do not need to work several jobs or split time between the hospital and their private practice.”* (P053)
	More time for patient consultations	*“In Sweden, a doctor has 30 min for a patient in an outpatient clinic and not 7–10 min like in Poland.”* (P037)
	Active involvement of nurses and social workers in patient care	*“The presence of social workers in hospitals who take over a certain part of the duties of doctors (which in Poland have to be performed by doctors alone or are not performed by anyone)”* (P013)

**Table 5:** The outcome of respondents’ efforts to implement changes to the healthcare or educational system in Poland.

Axial Code	Theme	No. (*n* = 19)
No or insignificant change	Little or no effect	13
	Conversations about differences with colleagues	2
	Ceased to convince other doctors	1
	Frustration	1
Reasons for lack of success in change	Most doctors accept the traditional system	1
	Opposition to changes in the profession	1
	Difficulty changing system that lasts for years	1
Successful change	Limited to my own approach to education/teaching	4
	Limited to my own approach to healthcare delivery	3

## Data Availability

The data used to support the findings in this study are available from the corresponding author upon request.

## Appendix A

**Table A1:** Outline of questions asked in the survey along with the numbers of respondents who answered them.

Questions Asked	No. of Respondents
1. What is your age? (open question)	99
2. What is your gender? (closed question)	104
3. What academic year did you participate in the ERASMUS exchange? (closed question)	109
4. Which university did you visit as part of the ERASMUS program? (closed question)	106
5. Which year of study were you in when you undertook your ERASMUS exchange? (closed question)	109
6. What area of medicine do you qualify, train in, or plan to do so? (closed question, more than one choice possible, including not intending to specialize).	106
7. Which of the following best describe your current professional role (more than one possible)? (closed question)	108
8. Since graduating, have you worked abroad in healthcare or university/research (if the answer is no, please go to question No. 11)? (Y/N closed question)	107
9. Please tell us more about your work abroad in healthcare or university/research (please select boxes that apply). - Temporary post (healthcare) - Permanent post (healthcare) - Training post (specialty training) - Locum - Teaching post - Research - Other (closed question)	28
10. Please tell us more about this post, e.g., when it began and will end, and what you hope to do afterward? (open question)	24
11. Why did you take part in the ERASMUS exchange? - to experience this unique opportunity to study abroad - out of curiosity - to improve the quality of my education - to improve language skills - to experience a different culture - for a change of routine - to get to know a different educational system - to improve my social life - to improve my prospects of further study abroad - to improve my prospects of getting a job abroad - to improve my prospects of further study in Poland - to improve my prospects of getting a job in Poland - for personal reasons - for research opportunities - to undertake the specific courses offered - other (closed question, more than one choice possible)	109
12. What do you feel were your major achievements during the ERASMUS visit? (open question)	62
13. How would you rate the importance of the benefits you gained from your ERASMUS visit? - social - touristic - language - educational - professional networking - organization skills other (Likert 1–5 closed question, 1 = no benefit; 5 = great benefit)	108
14. How would you rate the importance of the social benefits you gained from your ERASMUS visit? - Learning teamwork - Making international contacts - Keeping international contacts (after ERASMUS) - Learning to live independently - Making new friends - Rich and interesting social life - Other (...) (Likert 1–5 closed question, 1 = no benefit; 5 = great benefit)	109
15. When choosing where to go, did language influence your choice? (Y/N closed question)	109
16. If yes, did you want to visit a place where you would need to speak: - a language you have been learning - a language completely new for you (closed question)	100
17. Did you speak your host country’s mother tongue? (Y/N closed question)	109
18. Which language did you use to speak with fellow students and staff? - their mother tongue - another language when possible (closed question)	108
19. How competent were you in communicating with fellow students and university staff? - fluent - little difficulty - moderate difficulty - severe difficulty (closed question)	109
20. Which language did you use to speak with patients [MCQ] - their mother tongue - another language when possible - always with the aid of an interpreter - I did not have contact with patients (closed question)	109
21. How competent were you in communicating with patients? - fluent - little difficulty - moderate difficulty - severe difficulty (closed question)	108
22. How did your professional foreign language skills change as a result of your ERASMUS experience? - significantly improved - little improved - not affected - became worse (closed question)	109
23. Did you note anything particularly different about the medical education system in the country you visited? If so, please describe. (open question)	76
24. Do you recall any particularly memorable educational events (good or bad) during your ERASMUS exchange? If so, please describe briefly below, indicating whether each was positive or negative for you? (open question)	61
25. How would you rate the competences of the host institution’s students’ when compared with that of Polish students from the home institution? - theoretical knowledge - practical skills (Likert 1–5 closed question, 1 = much worse; 5 = much better)	102
26. Following on from your ERASMUS educational experiences, have you changed your approach to learning? (open question)	74
27. Following on from your ERASMUS educational experiences, are there changes you would like to see introduced to the Polish medical education system or methods? (open question)	79
28. Did your ERASMUS visit influence your career plans? (Y/N closed question)	104
29. If yes, please tell us whether, as a result of ERASMUS, you were less or more likely: - to undertake postgraduate studying and training abroad? - to look for a permanent job abroad? (Likert 1–5 closed question)	76
30. Did you note anything particularly different about the healthcare system in the country you visited? (open question)	65
31. Following on from your ERASMUS healthcare experiences, have you changed your approach to clinical practice? (open question)	67
32. Following on from your ERASMUS healthcare experiences, are there changes you would like to see introduced to the Polish healthcare system? (open question)	67
33. Have you tried to implement changes to the Polish educational or healthcare systems or convince others to do so as a result of your ERASMUS experience? (Y/N closed question)	104
34. If so, what was the outcome of your efforts to implement change? (open question)	19
35. Do you have any further comments about your ERASMUS experiences or the effect the visit has had on you? (open question)	39
